# Nanomaterials and continuous wave laser-based efficient desorption for atmospheric pressure mass spectrometric imaging of live hippocampal tissue slices†

**DOI:** 10.1039/c8ra00038g

**Published:** 2018-02-20

**Authors:** Jae Young Kim, Eun Seok Seo, Hee Jin Lim, Hyunmin Kim, Ji-Won Park, Hyeon Ho Shin, Dong-Kwon Lim, Dae Won Moon

**Affiliations:** Department of New Biology, Daegu Gyeongbuk Institute of Science and Technology (DGIST) Daegu Republic of Korea dwmoon@dgist.ac.kr; Companion Diagnostics and Medical Technology Research Group, Daegu Gyeongbuk Institute of Science and Technology (DGIST) Daegu Republic of Korea; Graduate School of Analytical Science and Technology (GRAST), Chungnam National University Daejeon Republic of Korea; KU-KIST Graduate School of Converging Science and Technology, Korea University 145 Anam-ro, Seongbuk-gu Seoul Republic of Korea dklim@korea.ac.kr

## Abstract

Micrometer-resolution mass spectrometric imaging of live hippocampal tissue is achieved with a highly efficient desorption of biomolecules using a 532 nm continuous wave laser and gold nanoparticles or graphene oxide as an energy transporter, which enables clear identification of the distributions of monoacylglycerol, adenine, cholesterol, sphingosine and ceramide.

## Abstract 2

Micrometer-resolution mass spectrometric imaging of live hippocampal tissue is achieved with a highly efficient desorption of biomolecules using a 532 nm continuous wave laser and gold nanoparticles or graphene oxide as an energy transporter.

## Graphical abstract

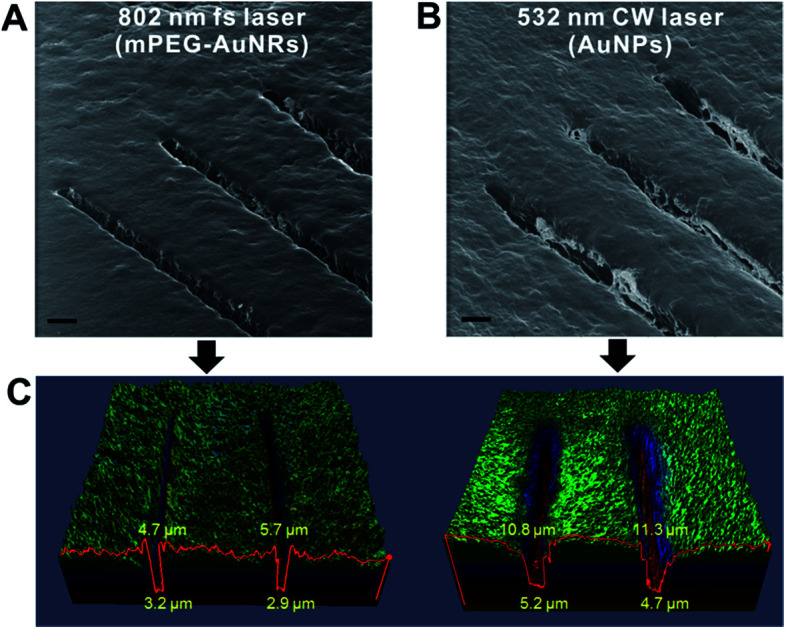

There are wide ranges of applications of the atmospheric pressure mass spectrometry (AP-MS) method to detect pesticides or explosive materials or for biological samples that should be analysed in an ambient environment to get more accurate information and/or for real-time analysis.^1–5^ AP-MS imaging can provide chemical and spatial information of molecules of interest, which is important for bioimaging applications. The AP-MS method is based on efficient desorption and ionization procedures under open-air atmospheric pressure and ambient temperature conditions.^6–8^ Although several methods, such as desorption electro-spray ionization, matrix-assisted laser desorption ionization, or laser ablation electrospray ionization, have been used to obtain MS imaging of biological samples, limited spatial resolution (several-tens-to-hundred micro-meters) and complex instrumental setup have been the main limitations of AP-MS imaging.^9–11^ The use of organic or inorganic nanomaterials as an efficient matrix for mass analysis is also a viable approach.^12–18^

Recently, we reported a high spatial resolution AP-MS imaging system with efficient desorption procedures by use of gold nanorods (AuNRs) and femtosecond (fs) pulsed laser oscillator, and subsequent ionization step with an aid of plasma system (termed AP-nanoPALDI MS system), which showed a micro-meter spatial resolution and detailed chemical information of live mouse hippocampal tissues.^19^ However, for more practical applications of AP-MS system, such as endoscopic platform for *in situ* cancerous cell detection in the clinic, a simple laser setup compatible with normal optical fiber is more desirable. The fs laser oscillator provides high energy sufficient to produce desorption of molecules, but the high peak power (several hundred kW) requires the use of the specific optical fiber and the distorted wave property in the optical fiber is another critical issues for cost-effective *in situ* endoscopic applications.^20–22^

In this work, we solved such a problem by using 532 nm-continuous wave (CW) laser for desorption. Although K.-C. Schäfer *et al.*, investigated a CW laser source for *in situ* realtime identification of biological tissues, it requires high output power (∼*W*) and visible region wavelength was not possible to produce efficient desorption.^23^ Therefore, the use of materials that can respond to a 532 nm laser is essential to facilitate the desorption process. We used spherical gold nanoparticles (AuNPs) as a light absorber to visible wavelength. We also investigated the use of carbon-based nanomaterials, such as graphene, that can respond to multiple wavelengths because of broad and strong light absorption property in the visible and NIR region of light, which can widen the availability of light source for sample desorption.

We used the previously developed AP-nanoPALDI MS system with a change of light source. The AP-MS system for this study is described in detail in ESI Note 1 and Fig. S1.† A 532 nm CW laser beam was introduced into the inverted microscopy by a dichromic beam splitter (NFD01-532-25x36, Semrock, USA) and was focused precisely on the specimen through the objective lens (Scheme 1). Because a visible light laser was employed as the light source for desorption, this configuration enabled both optical imaging monitoring and laser desorption in biological samples through the same objective lens. All the MS images in this study were obtained using a 20× objective lens (NA = 0.45; LUCPlanFLN 20X, Olympus, Japan) to focus the laser beam. The neutral molecule substances desorbed by the focused CW laser compulsorily meet the helium plasma medium by a separate plasma ionization source, and some were ionized by metastable helium atoms with excitation energies of 19.8 eV.

**Scheme 1:**
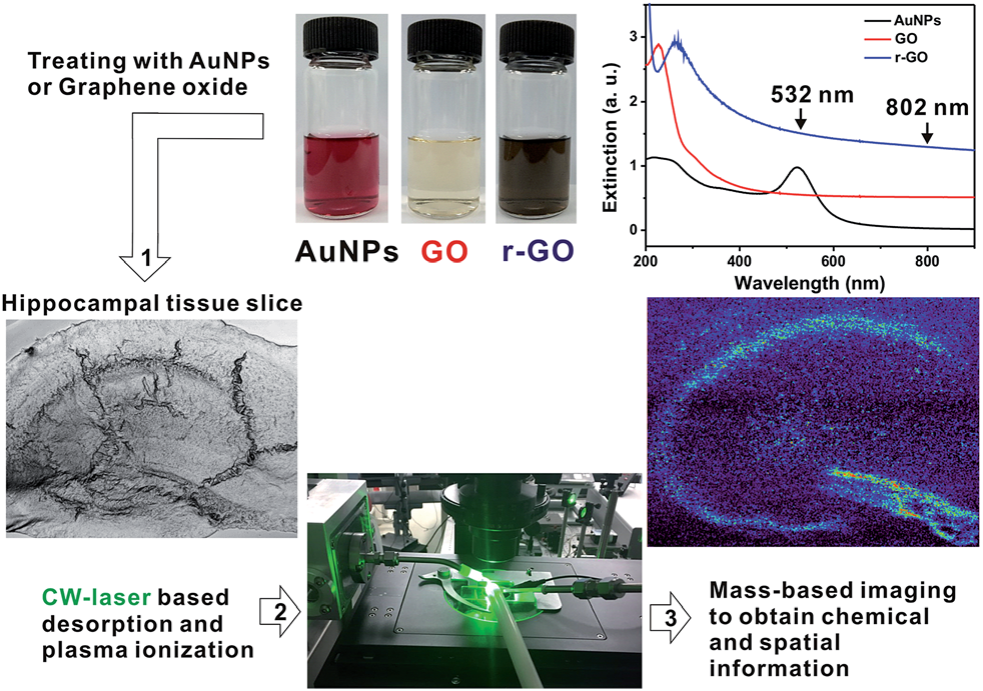
Visible-light-absorbing nanomaterials and CW-laser-based mass spectrometry imaging system for live hippocampal tissue slice. (1) Incubating nanomaterials on tissue, (2) CW-based desorption and ionization with laser and plasma, (3) mass-based spectrometry imaging process.

Adult-mouse (7 week old, male, C57BL/6) hippocampal tissue slices were chosen for tissue imaging because of the distinctive structures and functions related with memories. Especially we focused on cornu ammonis 1 (CA1), cornu ammonis 3 (CA3), and dentate gyrus (DG), since the connections among these three regions play an important role in consolidating information of short-term memories to long-term memories and spatial navigation.^24–26^ For AP-MS imaging, first, the fresh tissue slice of adult-mouse hippocampus (200 μm thickness) was incubated with AuNPs (0.5 nM, 20 nm) or nanosized graphene oxide (GO) (<100 nm, 0.2 mg mL^−1^) in the artificial cerebral spinal fluid (ACSF) for 1 h (see ESI Notes 2 & 3†). After incubation, the tissue was washed 10 times with ACSF, and then placed tissue on the scanning stage for imaging. Transmission electron microscopy analysis showed the relatively uniform distribution of AuNPs on the tissue surface (Fig. S2†).

To examine the possibility of CW laser for efficient desorption, we performed single line scanning for the tissue treated with AuNPs (20 nm, *λ*_max_ = 520 nm) and applied a 532 nm CW laser (300 mW, 20×, 0.45 NA objective lens). The results were compared with that of a previous fs laser-based AP-MS system. The helium ion microscopy (HIM) images in Fig. 1A scanned with the fs-based laser AP-MS system show narrow bottom and top widths (3.2, and 2.9 μm, and 4.7 and 5.7 μm, respectively) of desorbed tissue area (Fig. 1C). The line scanned area with the CW-based laser AP-MS system also shows successful desorption with a micrometer resolution (bottom width 5.2, 4.7 μm, top width 10.8, 11.3 μm) indicating the possibility of using the CW-based laser for AP-MS system (Fig. 1C).

**Fig. 1:**
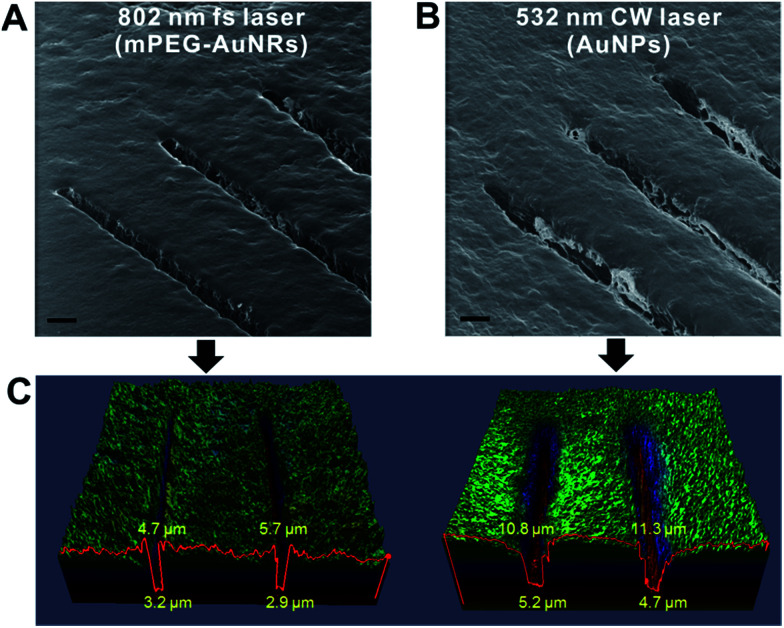
Helium ion microscopy (HIM, Orion NanoFab, Carl Zeiss, USA) images of hippocampal tissue after line scanning with 802 nm fs laser (A), or with CW laser (B). (C) Confocal fluorescence images of both samples from the confocal laser scanning microscope (LSM-700, Carl Zeiss, Germany). Scale bar in (A) and (B) is 10 μm.

The use of nanomaterial and selection of sufficient input laser power were critical factors for successful desorption and reliable MS imaging. No such clear desorption could be found in case of the absence of nanomaterials (Fig. 2A and S3†). Only a small part of tissue surface was desorbed when CW laser was applied on the tissues without treating nanomaterials. The tissues treated with AuNPs show uniform and reproducible desorption patterns with application of CW laser with input power higher than 200 mW (Fig. 2B). Applying less than 200 mW is not desirable because of non-uniform desorption patterns shown in Fig. 2B (100 mW, 75 mW). Non-uniform desorption can cause significant defects in MS imaging. The tissue treated with GO solution shows very uniform and sharp desorption, even with a 100 mW input laser application. Interestingly, in spite of stronger light absorption properties of r-GO, the tissue sample treated with r-GO solution shows non-uniform and inefficient desorption patterns compared with the GO-treated tissue sample. As shown in Fig. 2D, applying 100 mW is not sufficient output power to produce uniform desorption. This is possibly due to decreased dispersity of r-GO in aqueous buffer condition and resulting non-uniform distribution of r-GO on tissue samples. In this regard, GO solution is the most desirable nanomaterial in terms of desorption.

**Fig. 2:**
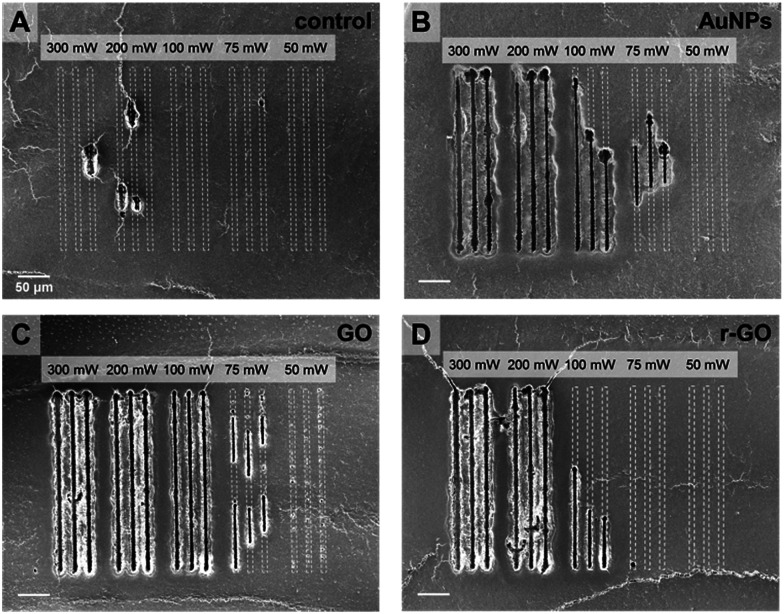
Helium ion microscopy (HIM) images of linear craters on hippocampal tissue slices, which desorbed by focused CW laser beam with various output powers and hippocampal tissue slice treated without nanomaterials (A), or with AuNPs (B), GO (C), and r-GO solution (D). (Scale bar = 50 μm).

Next, we applied the CW-laser AP-MS system to obtain hippocampal tissue imaging with a narrow pixel size of 4.2 μm × 5.0 μm covering an area of 1800 μm × 1500 μm (433 × 300 pixels) not to make dead pixel in the image. The actual resolution for the image will be about 10 μm based on minimum desorption area shown in Fig. 1 & 2. Total acquisition time was 350 min. The scanning conditions for an accurate timing among scanning stage, laser trigger, and signal acquisition was described in detail in ESI Note 4 and Fig. S4.† After removing the plasma background, MS spectra of more than 200 specimens were obtained, and some of the detected ions were identified as metabolites, lipids, and their derivatives by a comparison of the measured masses and the calculated chemical formulas (Fig. S5†). Several saturated and unsaturated monoacylglycerol (MAG) ions could be identified based on chemical formulas, such as MAG 16:0, MAG 18:0, MAG 16:1, MAG 18:1, and MAG 18:2. Other lipid and metabolite ions were identified, such as adenine, cholesterol, sphingosine, sphinganine, and ceramide 18:0.

By transforming each MS spectrum with BioMAP software (Novartis Institutes for BioMedical Research, Cambridge, USA), MS-based images for hippocampal tissue were obtained as shown in Fig. 3A. After MS analysis with CW laser, the scanned area showed complete destruction indicating efficient desorption and ionization process occurred in this area. The MS images with selected ion-species for MAGs (16:1, 16:0, 18:1, 18:0), adenine, cholesterol, sphingosine and ceramide are displayed in Fig. 3B, C.

**Fig. 3:**
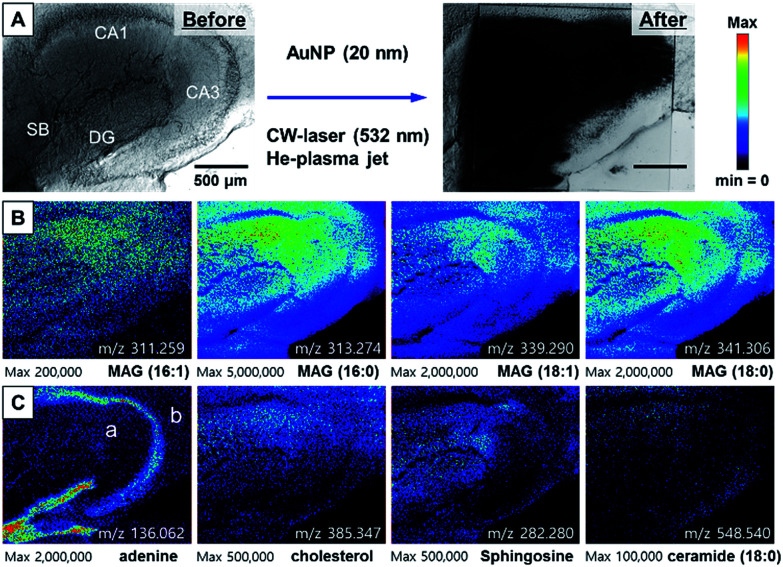
(A) The photo of hippocampal tissue before and after MS analysis with the treatment of AuNPs and 532 nm CW laser (300 mW), (B) ion images for MAGs, (C) ion images for adenine, cholesterol, sphingosine, and ceramide 18:0. In the adenine ion image at *m*/*z* = 136.062, (a) represents an apical dendritic area and (b) represents a basal dendritic area.

The images constructed with selected ion species exhibited mostly similar spatial distribution with that of our previously reported NIR (802 nm) fs laser and mPEG-AuNRs based ion images displayed in Fig. S6.† The image obtained with adenine *m*/*z* = 136.062 clearly shows the distribution of soma (cell body) of neurons in the tissue specimen that is commonly observed in nucleus is concentrated in the DG (Fig. 3C). Moreover, the ion image of adenine can clearly distinguish the apical and basal dendritic areas, located on the inside and outside of the soma location, respectively (see (a) and (b) in ion image of adenine at *m*/*z* = 136.062 in Fig. 3C). As shown in Fig. 3B and C, MAGs, sphingosine, and cholesterol were found to be most abundant in the apical dendritic area of CA1 of the hippocampus tissue. These high-resolution ion images can be used to study the molecular differences between the apical and basal dendrites of the neurons at the tissue level.

In addition to the use of AuNPs as an efficient light absorber of 532 nm laser, GO nanosheet is also an excellent nanomaterial for efficient desorption, as demonstrated in Fig. 2C and S3.† We obtained the AP-MS imaging of the hippocampal tissue slice treated with GO solution (0.2 mg mL^−1^) and CW laser (Fig. 4). The scanned area shows the completely and uniformly desorbed area. As for this MS analysis method, since the ionization occurs in a separate plasma source after desorption procedure by laser, the obtained mass spectra were not significantly different from those obtained with AuNPs. The MS images constructed with MAG mass spectrum showed uniform distribution of MAG ions in the tissues (Fig. 4B). The adenine distributions in this mass analysis are identical with the image obtained with AuNPs in Fig. 3C. MAGs were found to be most abundant in the apical dendritic area of CA1 of the hippocampus tissue, and ceramide 18:0 and sphingosine were abundant in the basal dendrite area as well as the apical dendrite area in the CA region of the tissue. Overall, the MS images obtained with GO and CW laser showed more reliable information on the spatial distribution of molecules, mainly because of uniform distribution of GO nanosheets on tissue specimen and efficient desorption. Since GO also absorbs the NIR light, the possibility of AP-MS analysis with NIR fs laser was also confirmed (Fig. S7†). The CA1 region in the tissue was successfully desorbed, showing clear corresponding MS images.

**Fig. 4:**
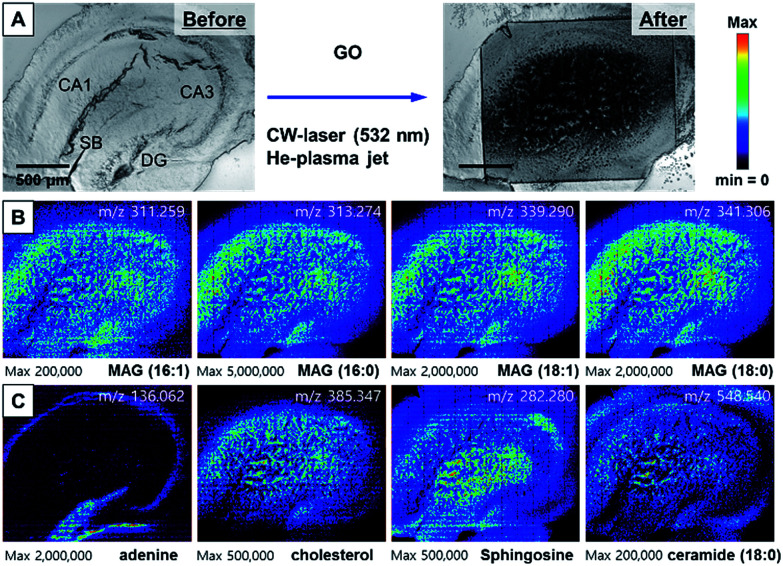
(A) The photo of hippocampal tissue before and after MS analysis with the treatment of GO solution and 532 nm CW laser (300 mW), (B) ion images for MAGs, (C) ion images for adenine, cholesterol, sphingosine, and ceramide.

These results indicate the strong potential of GO for wide ranges of light sources for efficient desorption.

To accurately compare the performance of r-GO for AP-MS imaging, we performed MS imaging after treating r-GO solution (0.2 mg mL^−1^) on the hippocampal tissue. Applying 300 mW CW laser was successful based on the scanned bright field image in Fig. 5A. The distribution of MAGs on apical and basal dendrites of hippocampal tissue slice was very similar with the results obtained with AuNPs (Fig. 5B). However, the spatial distribution of mass ion is not regular. The most distinctive result is the distribution of MAG (16:0) (in Fig. 5B) compared with the same ion images in Fig. 3B, 4B. The distribution of the MAG (16:0) ion spectrum in the images obtained with AuNPs or GO was quite uniform. Although the distribution of the adenine mass spectrum was visible, the signal intensity was weaker compared with the images obtained with AuNPs or GO (Fig. 5C). The low solubility and non-uniform distribution of r-GO can be assumed the main reasons of these observed irregular distributions of selected mass ions.

**Fig. 5:**
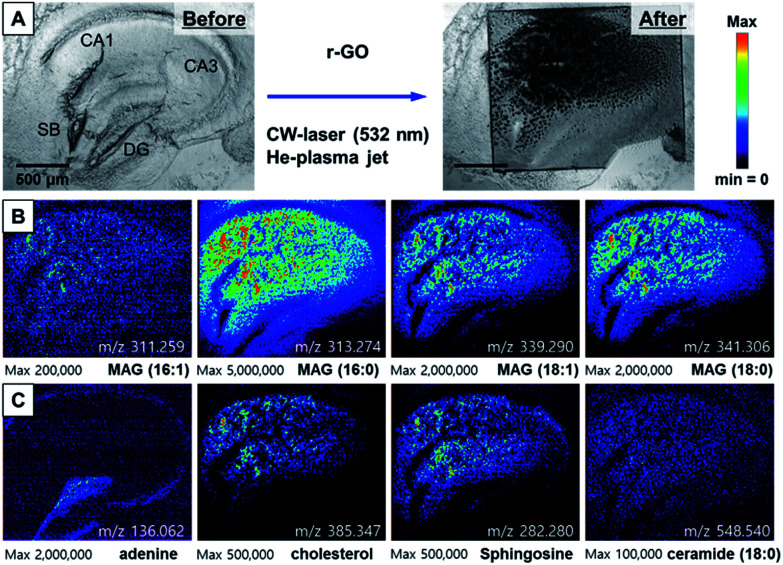
(A) The photo of hippocampal tissue before and after MS analysis with the treatment of r-GO solution and 532 nm CW laser (300 mW), (B) ion images for MAGs, (C) ion images for adenine, cholesterol, sphingosine, and ceramide.

All these results indicate that the efficient desorption of molecules on the wet-state hippocampal tissue for AP-MS imaging is possible by use of nanomaterials such as AuNPs or carbon nanomaterials. In addition, the CW-laser is also an excellent light source for AP-MS imaging, which can greatly expand the ranges of application of the AP-MS-based analysis method in real-time mass analysis with endoscopic devices.

In summary, we demonstrate that CW-laser based AP-MS imaging is possible with the use of nanomaterials. The incubation of tissues with AuNPs or nanosized graphene oxides (<100 nm) was essential to induce efficient desorption of wet-state hippocampal tissue. Because of excellent dispersity of GO in aqueous buffer solution, GO was found to be the most desirable nanomaterials for efficient desorption to increase the sensitivity and reproducibility of the AP-MS imaging system. The successful AP-MS imaging with CW-laser system highlights the advancement of AP-MS imaging technology into future clinical applications. The CW-based AP-MS system can identify lipidomic and metabolic observations in live tissues or detect pathogenic tissues, and opens a new approach to MS endoscopy for *in vivo* mass spectrometry based diagnosis.

## Conflicts of interest

There are no conflicts to declare.

## Supplementary Material

RA-008-C8RA00038G-s001
