# Supplementary material for: Nanomaterials and continuous wave laser-based efficient desorption for atmospheric pressure mass spectrometric imaging of live hippocampal tissue slices
Source: RSC Adv. 2018 Feb 20;8(15):8021–5. doi: 10.1039/c8ra00038g (PMC9078475; doi:10.1039/c8ra00038g)
Supplement: RA-008-C8RA00038G-s001 [file RA-008-C8RA00038G-s001.pdf]

Electronic Supplementary Material (ESI) for RSC Advances.
This journal is © The Royal Society of Chemistry 2018

Supplementary Online Information for

Nanomaterials and Continuous Wave Laser-Based Efficient Desorption for Atmospheric Pressure Mass Spectrometric Imaging of Live Hippocampal Tissue Slice

Jae Young Kim, Eun Seok Seo, Hee Jin Lim, Hyunmin Kim, Ji-Won Park, Hyeon Ho Shin, Dong Kwon Lim*, and Dae Won Moon*

Supplementary Notes

1. AP-nanoPALDI mass spectrometry imaging system with visible CW lasers

The AP-nanoPALDI MS system consists of a mass analyzer, a sampling stage, a 532-nm continuous wave (CW) laser, an atmospheric pressure (AP) plasma device, and airflow-assisted ion transport equipment, as shown in Fig. S1(A).

In order to optically monitor the biological sample and decide the analysis region, a stage of an inverted optical microscope (IX73, Olympus, Japan) was used as a sampling stage. The diode-pumped solid-state laser system (MGL-III-532, CNI Optoelectronics Tech, China) generates CW laser light with an output power of 300 mW at 532 nm. The CW laser beam was introduced into the rear port of the inverted microscope. A dichroic beam splitter (NFD01-532-25x36, Semrock, USA) allowed optical imaging monitoring and laser desorption of a biological specimen simultaneously without a separate objective lens, as shown in Fig. S1(B). For precise positioning of a region of interest (ROI) at the micrometer level and sample moving at a constant velocity for raster scanning, a programmable motorized XY scanning stage (AS-MIX73-C, iNexus, South Korea) was mounted on the inverted optical microscope. The nonthermal AP helium plasma jet device, consisting of a quartz tube (2 mm ID and 3 mm OD) with discharge gases and electrodes, formed a helium plasma medium above the sample. The sinusoidal voltage (5 kV_p and a frequency of 27 kHz) was applied and a high-purity helium gas (HP grade; 99.999%) with a gas flow rate of 0.5 slm was used for helium plasma generation. The neutral molecules desorbed by the focused laser inevitably meet the helium plasma medium by arranging two desorption/ionization sources, and some were ionized by metastable helium atoms with excitation energies of 19.8 eV. The additional pumping system was installed in the mass

analyzer for effective transport of molecules and ions. The airflow-assisted ion transfer equipment consisted of an ion transfer tubing, a chamber, and a diaphragm pump. The pump generated airflow inside the ion transfer tube toward the MS inlet. This equipment, coupled with the mass spectrometer, successfully transfers ions to the MS inlet under atmospheric pressure conditions where no pressure difference exists.

2. Preparation of nanosized GO and r-GO

GO was prepared by a modified Hummer′s method. A 9:1 mixture of concentrated H_2SO_4/H_3PO_4 (360:40 mL) was added to a mixture of graphite flakes (3.0 g, 1 wt equiv). With stirring and cooling in an ice bath, KMnO4 (18.0 g, 6 wt equiv) was slowly added into the reaction mixture, producing a slight exotherm to 35−40 °C. The reaction was then heated to 50 °C and stirred for 12 h. The reaction was cooled to room temperature and poured onto ice (400 mL) containing 30% H_2O_2 (3 mL). The solution was then filtered through a metal U.S. Standard testing sieve (W.S. Tyler, 300 μm). The filtrate was centrifuged (8,000 rpm for 2 h), and the supernatant decanted. The remaining material was then washed in succession with 200 mL of water, 200 mL of 30% HCl, 200 mL of ethanol, and water again until solution pH reached 5.0–6.0. The resulting suspension was filtered with a PTFE membrane with a 0.45-μm pore size and then lyophilized to produce a fluffy GO powder.

GO powder (5.0 mg) was dissolved in triple-distilled water (> 18 MΩ, 10 mL), then exfoliated by prolonged sonication (35% amplitude, 500 W, 4 hr) until the entire size distribution was below 150 nm, and then centrifuged (18,000 rpm, 20 min) to remove precipitates (un-exfoliated large GO sheets). The supernatant was analyzed by dynamic light scattering (DLS). DLS analysis (0.05 mg/mL) showed a narrow size distribution (average 60 nm) of GO sheets

after centrifugation. Importantly, the nanosized GO sheets were freely dispersible in water without aggregation.

To prepare r-GO, solution-based reduction with hydrazine in the presence of trace amount of NH_4OH (28%, w/w) was applied to produce reduced state of graphene oxide (r-GO), which confirmed with UV-Vis and FT-IR spectra.

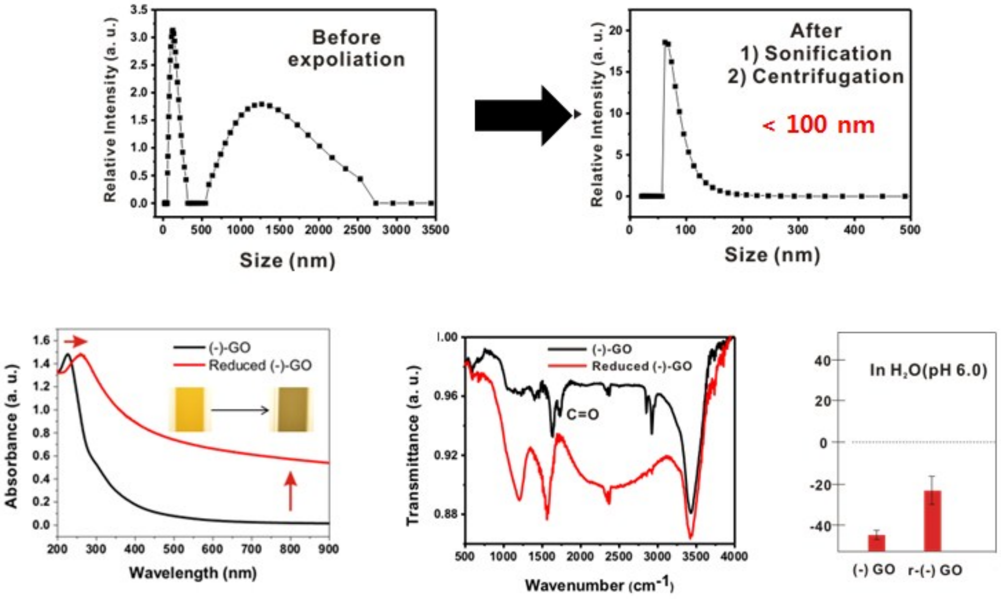

3. Specimen preparation of mouse hippocampal tissue slice

Short-term living tissue slices are well-established in pseudo-two-dimensional models for research into neuro-, patho-, and electro-physiologies. Thus, the sample preparation was similar to that of stretch-activated ion channels (SAC) or multielectrode arrays (MEAs) studies. Male 7-week-old C57BL/6 mice were purchased from Koatech (Pyeongtaek, South Korea) and housed in pathogen-free animal facilities. All experiments were approved and performed in accordance with the Daegu Gyeongbuk Institute of Science & Technology (DGIST, South Korea) guidelines for animal use and care.

After sacrificing and brain extraction, the hippocampus was isolated and transversely sliced at a thickness of 200 μm with a tissue chopper (McLlwain tissue chopper, Cavey Laboratory Engineering, UK). Slices were aerated with oxygenated sucrose artificial cerebral spinal fluid (sACSF) containing 124 mM NaCl, 2.5 mM KCl, 1.25 mM KH_2PO_4, 26 mM $NaHCO_3$, 2 mM $MgSO_4$, 2.5 mM $CaCl_2$, 10 mM D-glucose, and 4 mM D-sucrose bubbled with 95% O_2/5% CO_2 using an aquarium bubbler at 32°C for 2 h. After 1 h aeration, the slices were submerged with 5 ml of ACSF solution with nanomaterials, such as citrate-AuNPs, graphene oxide nanosheets, and reduced graphene oxide nanosheets. After 1 h incubation with the nanomaterials, the hippocampal slices were washed 10 times and placed on 0.1% polyethylenimine (PEI, in 25 mM borate buffer)-coated slides to facilitate tissue adhesion. Since these tissue specimens do not need to be completely dried, the analysis can be started within 20 min. It is noteworthy that even though the mice were sacrificed for sample preparation in this study, the extracted hippocampal tissue slice was certainly viable until MS analysis began and could be cultured for several weeks.

4. Assembled scanning system for an accurate timing among scanning stage, laser trigger, and signal acquisition

A programmable motorized X-Y scanning stage was mounted on the inverted optical microscope for precise manual positioning at the micrometer level and a sample moving at a constant velocity for raster scanning. Importantly, the raster scanning should be performed by only moving this sample stage with being kept the fixed positions of the ion transfer tube, the atmospheric pressure plasma jet device, and the laser focus. With this arrangement, samples can be positioned within an area of 70 × 50 mm and moved with a maximum resolution of 0.5 μm for providing fresh specimen. The stepper motors are externally controlled via both a joystick and a motion control program, and all scanning parameters, such as X-Y coordinates, scan speed, scan direction, interruption time, and number of scan, were programmable using the customized stage control software.

In addition, its motion control program relayed logic signals to the scanning stage, the mass analyzer, and the optical shutter for lasers, simultaneously as shown in Fig. S4. This allowed the scanning process by the laser desorption to be synchronized with the MS data acquisition with good accuracy. The sample stage was line scanned in the x-direction, and fed in the y-direction for MS imaging. Each single line scan along the x-direction was saved as one data file. Using the sequence mode of the data acquisition program in this mass analyzer, several hundred data files could be achieved in one experiment. The data files of multiple scan lines from the analytic area must be assembled in one data file for the MS image plotted against the x and y coordinates for a set of line scans in the x direction and fed in the y-direction.

Supplementary Figures

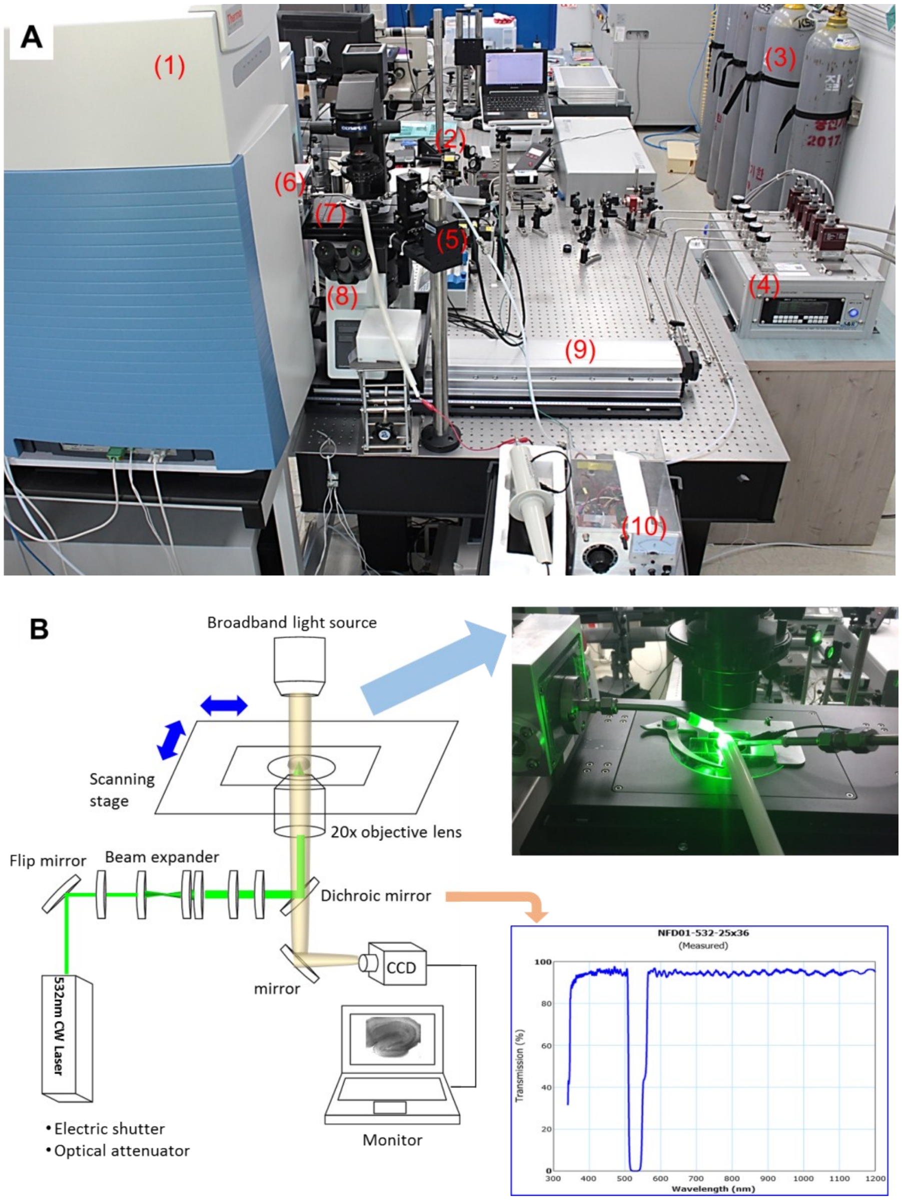

Figure S1. (A) A picture of the whole system for the AP-nanoPALDI mass spectrometry imaging employed in this study. The proposed system mainly consisted of mass analyzer, sampling stage, fs lasers system, atmospheric pressure plasma system, and airflow-assisted ion transport equipment; (1) QE Orbitrap mass spectrometer, (2) 532nm CW laser system, (3) gas cylinders, (4) 5 ch. gas flow controller, (5) nonthermal atmospheric pressure plasma jet, (6) airflow-assisted ion transfer system, (7) auto scanning stage, (8) inverted microscope, (9) long drive scope stage for microscope shift, and (10) high voltage driving circuit and probe. (B) Different light paths between visible and monochromatic light at 532 nm using dichroic mirror in the inverted optical microscope. This feature allows the 532-nm monochromatic light to be reflected towards the target while the other visible light passes through.

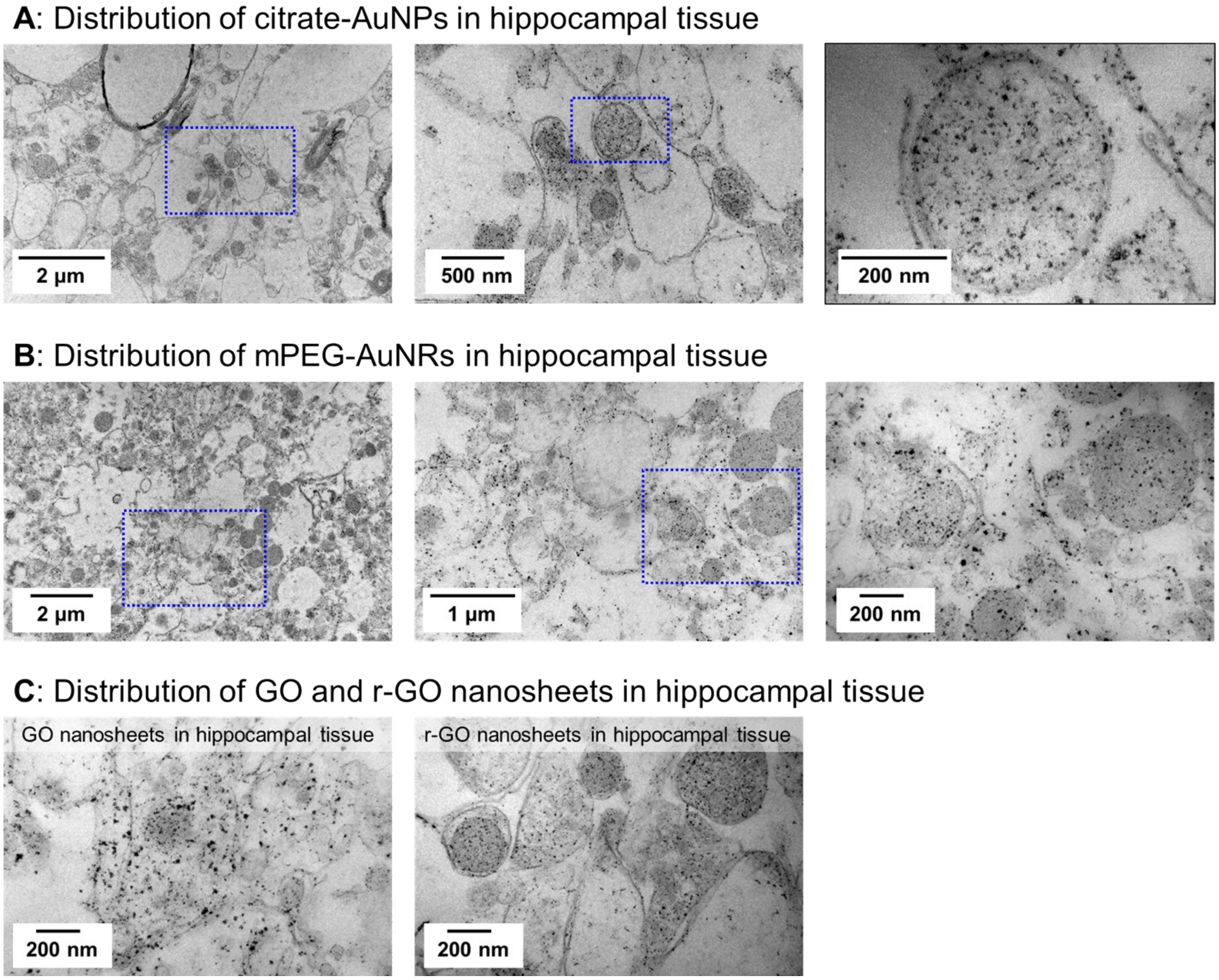

Figure S2. TEM image of hippocampal tissue incubated with (A) citrate-AuNPs, (B) mPEG-AuNRs, and (C) GO and r-GO nanosheets.

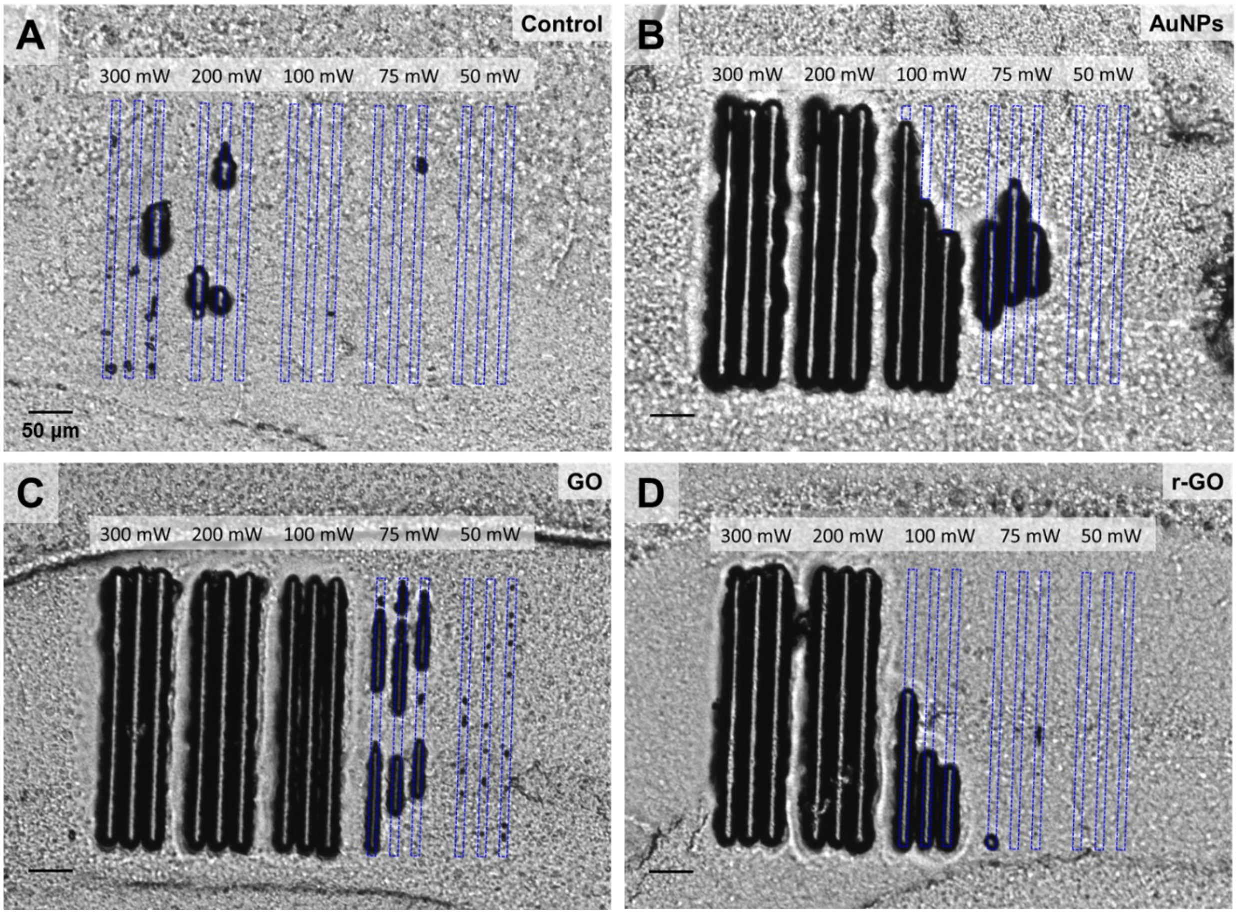

Figure S3. Optical microscopy images of linear craters on hippocampal tissue slices, which desorbed by focused CW laser beam with various output powers; (A) hippocampal tissue slice without any nanoparticles (control), hippocampal tissue slices with citrate-AuNPs (B), graphene oxide nanosheets (C) and reduced graphene oxide nanosheets (D).

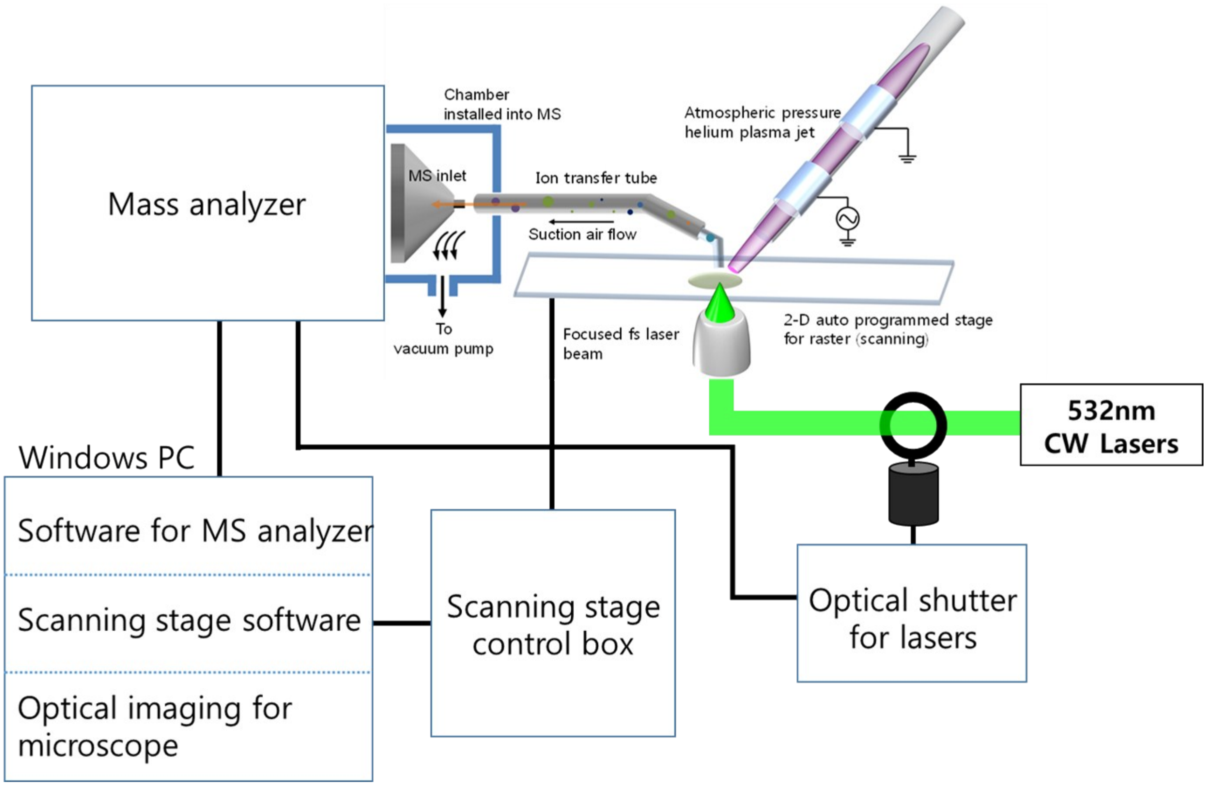

Figure S4. Schematics of assembled scanning system setup for MS imaging including experiment timing sequence of moving platform, laser trigger, and signal acquisition.

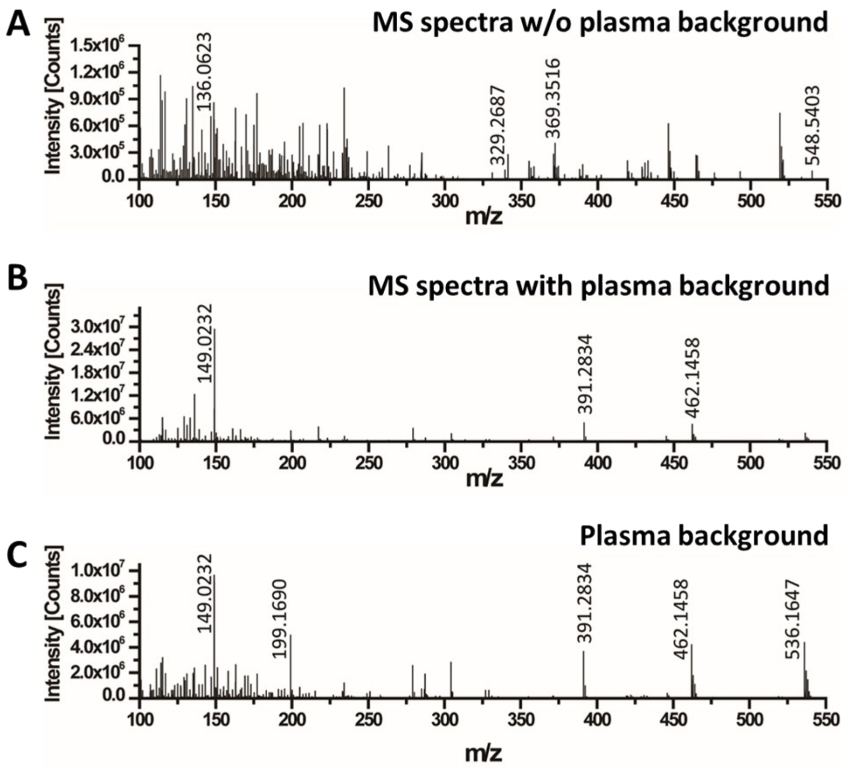

D Class	Compound	Measure m/z	Theoretical m/z	error (ppm)	Formula	Species
**Sterol lipid**	**Cholesterol**	369.3516	369.3515	0.27	$C_{27}H_{45}$	$[M+H-H_2O]^+$
		385.3471	385.3464	1.82	$C_{27}H_{45}O$	$[M-H]^+$
**Glycerolipid**	**MAG 16:1**	311.2587	311.2581	1.93	$C_{19}H_{35}O_3$	$[M+H-H_2O]^+$
		329.2687	329.2686	0.30	$C_{19}H_{37}O_4$	$[M+H]^+$
	**MAG 16:0**	313.2743	313.2737	1.92	$C_{19}H_{37}O_3$	$[M+H-H_2O]^+$
		331.2850	331.2843	2.11	$C_{19}H_{39}O_4$	$[M+H]^+$
	**MAG 18:2**	337.2737	337.2737	0.00	$C_{21}H_{37}O_3$	$[M+H-H_2O]^+$
		355.2849	355.2843	1.69	$C_{21}H_{39}O_4$	$[M+H]^+$
	**MAG 18:1**	339.2901	339.2894	2.06	$C_{21}H_{39}O_3$	$[M+H-H_2O]^+$
		357.3012	357.2999	3.64	$C_{21}H_{41}O_4$	$[M+H]^+$
	**MAG 18:0**	341.3057	341.3050	2.05	$C_{21}H_{41}O_3$	$[M+H-H_2O]^+$
		359.3151	359.3156	-1.39	$C_{21}H_{43}O_4$	$[M+H]^+$
**Sphingolipid**	**Ceramide 18:0**	548.5403	548.5401	0.36	$C_{36}H_{70}NO_2$	$[M+H-H_2O]^+$
	**Sphingosine**	282.2797	282.2791	2.13	$C_{18}H_{36}NO$	$[M+H-H_2O]^+$
		300.2908	300.2897	3.66	$C_{18}H_{38}NO_2$	$[M+H]^+$
	**Sphinganine**	284.2954	284.2947	2.46	$C_{18}H_{38}NO$	$[M+H-H_2O]^+$
		302.3062	302.3054	2.65	$C_{18}H_{40}NO_2$	$[M+H]^+$
**Adenine**		136.0623	136.0617	4.41	$C_5H_6N_5$	$[M+H]^+$
**Phosphocholine**		184.0753	184.0733	10.87	$C_5H_{15}NO_4P$	$[M]^+$

Figure S5. (A) Plasma background subtracted mass spectra of a mouse hippocampal tissue slice in the positive ion mode. (B) AP-MS spectra from a mouse hippocampal tissue slice with helium plasma background. (C) AP-MS spectra from AP helium plasma background. (D) Assigned lipids, metabolites, and derivatives from mouse hippocampal tissue slice in the positive ion mode.

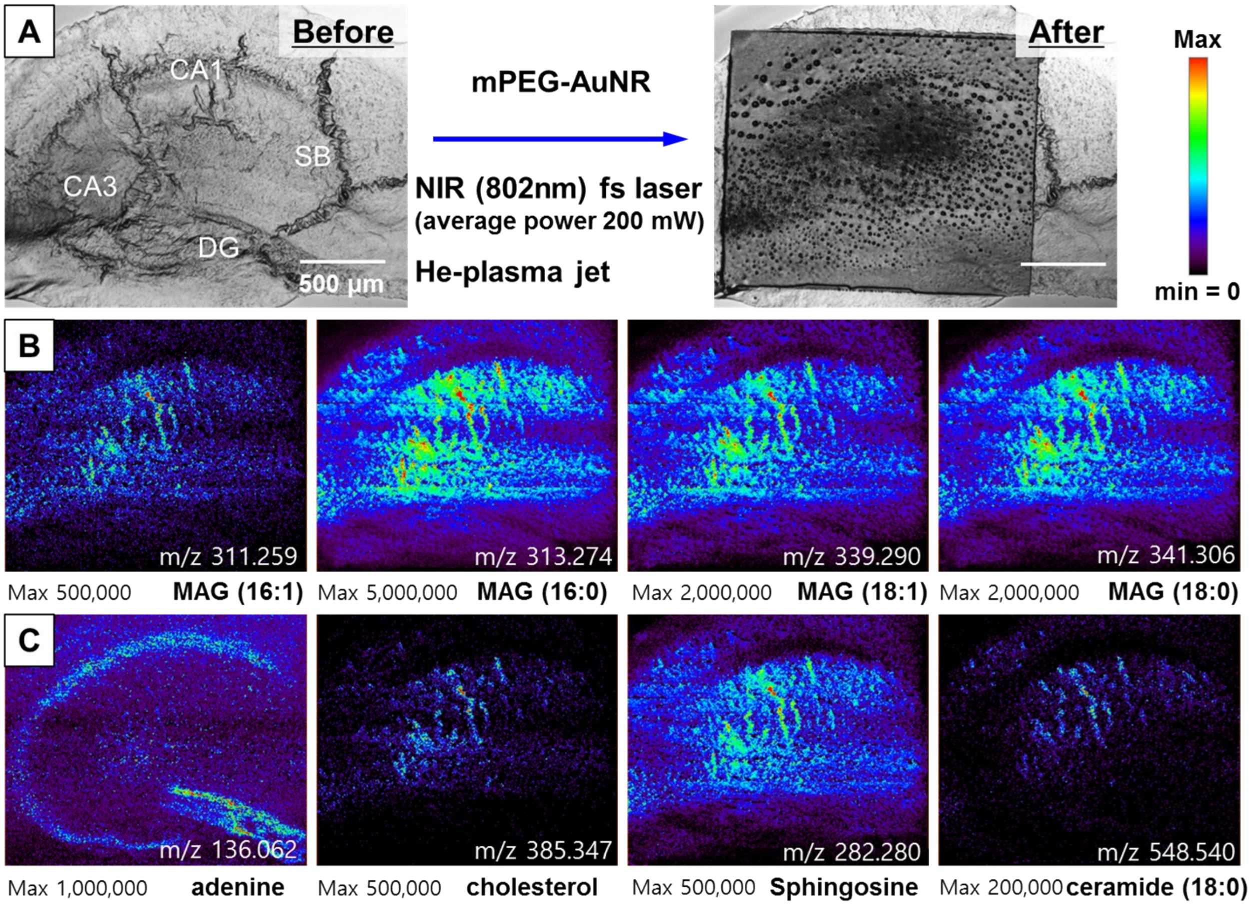

Figure S6. (A) The photo of hippocampal tissue before and after MS analysis with the treatment of mPEG-AuNRs and 802-nm fs laser (200 mW), (B) ion images for MAGs, (C) ion images for adenine, cholesterol, sphingosine, and ceramide.

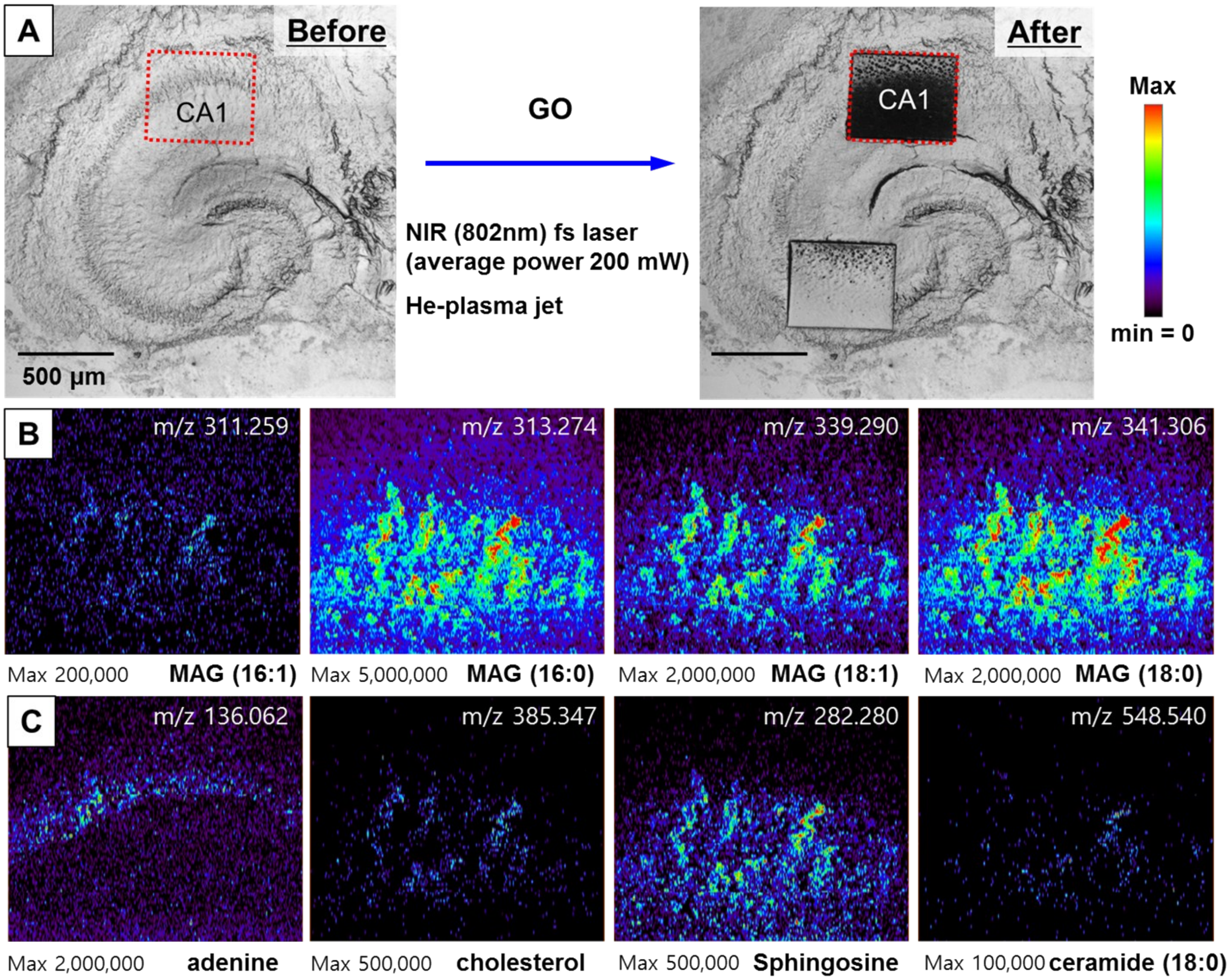

Figure S7. (A) The photo of hippocampal tissue before and after MS analysis with the treatment of GO and 802-nm fs laser (200 mW), (B) ion images for MAGs, (C) ion images for adenine, cholesterol, sphingosine, and ceramide.
